# Oxidative stress enhances the expression of IL-33 in human airway epithelial cells

**DOI:** 10.1186/s12931-018-0752-9

**Published:** 2018-03-27

**Authors:** Hiroyuki Aizawa, Akira Koarai, Yutaka Shishikura, Satoru Yanagisawa, Mutsuo Yamaya, Hisatoshi Sugiura, Tadahisa Numakura, Mitsuhiro Yamada, Tomohiro Ichikawa, Naoya Fujino, Masafumi Noda, Yoshinori Okada, Masakazu Ichinose

**Affiliations:** 1 Department of Respiratory Medicine, Tohoku University Graduate School of Medicine, 1-1 Seiryo-machi, Aoba-ku, Sendai, 980-8574 Japan; 2 Department of Advanced Preventive Medicine for Infectious Disease, Tohoku University Graduate School of Medicine, 1-1 Seiryo-machi, Aoba-ku, Sendai, 980-8575 Japan; 3 Department of Thoracic Surgery, Institute of Development, Aging and Cancer, Tohoku University, 4-1 Seiryo-machi, Aoba-ku, Sendai, 980-8575 Japan

**Keywords:** COPD, Exacerbation, IL-33, Oxidative stress, Viral infection

## Abstract

**Background:**

Interleukin-33 (IL-33) is a cytokine belonging to the IL-1 family, and its possible involvement in the pathophysiology of COPD and viral-induced exacerbations has been demonstrated. IL-33 has been shown to be increased in the airway epithelial cells from COPD patients, but the regulating mechanism of IL-33 expression in airway epithelial cells remains largely unknown. In the current study, we examined whether oxidative stress, which participates in the pathogenesis of COPD, affects the expression of IL-33 in airway epithelial cells and also evaluated the effect during viral infection.

**Methods:**

The involvement of oxidative stress in the expression of IL-33, and its signal pathway was examined after stimulation with hydrogen peroxide (H_2_O_2_), with or without stimulation by polyinosinic-polycytidylic acid [poly (I:C)], a synthetic analogue of dsRNA that mimics viral infection, or rhinovirus infection in NCI-H292 cells and primary human bronchial epithelial cells (HBECs). In addition, the effect of antioxidant, *N*-acetylcysteine (NAC) in the expression of IL-33 was compared between HBECs from healthy subjects and those from COPD patients.

**Results:**

Treatment with H_2_O_2_ significantly potentiated IL-33 expression in NCI-H292 cells, and the potentiation was reversed by NAC treatment. Mitogen-activated protein kinase (MAPK) inhibitors, but not nuclear factor-kappa B inhibitors, also significantly decreased the H_2_O_2_-potentiated IL-33 expression. In addition, H_2_O_2_ significantly potentiated the poly (I:C)- or rhinovirus-stimulated IL-33 expression. In HBECs from healthy subjects, H_2_O_2_-potentiated IL-33 expression and its reversal by NAC was also confirmed. Under the condition without H_2_O_2_-stimulation, treatment with NAC significantly decreased the expression of IL-33 in HBECs from COPD patients, but not in those from healthy subjects.

**Conclusions:**

These results demonstrate that oxidative stress involves in the expression of IL-33 in airway epithelial cells via MAPK signal pathway and it augments IL-33 expression during viral infection. This mechanism may participate in the regulation of IL-33 expression in airway epithelial cells in COPD and the viral-induced exacerbations. Modulation of this pathway could become a therapeutic target for viral-induced exacerbations of COPD.

**Electronic supplementary material:**

The online version of this article (10.1186/s12931-018-0752-9) contains supplementary material, which is available to authorized users.

## Background

Chronic obstructive pulmonary disease (COPD) was ranked sixth as a cause of death in 1990, but it is currently the third leading cause of death in the world [1]. Exacerbations of COPD triggered by respiratory infections or environmental pollutants cause an acute deterioration in airway inflammation and lung function, and also lead to an increase in mortality and healthcare costs [2–5]. The prevention of exacerbations remains a key therapeutic goal, but current treatments have only modest benefits and considerable adverse effects. Therefore, since new treatments are urgently needed, a better understanding of the mechanisms of COPD exacerbations is needed.

Interleukin-33 (IL-33) is a cytokine belonging to the IL-1 family, which includes IL-1α, IL-1β and IL-18. It is mainly a nuclear protein in several types of cells including endothelial cells, epithelial cells and fibroblasts [6, 7]. Once released into the extracellular compartment in response to tissue damage or inflammation, extracellular IL-33 has been shown to act as a potent inducer of inflammation [6, 7]. Especially, IL-33 promotes T helper 2 cell (Th2) immunity, which leads to allergic pathological changes via ST2 receptor, a member of the IL-1/Toll-like receptor family [8]. IL-33 has been extensively characterized as functionally important in Th2-associated inflammatory diseases including asthma, atopic dermatitis and helminth infections [6]. Although higher expression of IL-33 has been reported in patients with asthma [9], it has also been demonstrated in bronchial epithelial cells from those with COPD, especially in a severe stage of COPD [10, 11]. In a COPD mouse model and in vitro airway epithelial cells, increases of IL-33 have been also shown, especially during viral infection or after cigarette smoke exposure [11]. Despite the increasing evidence suggesting the contribution of IL-33 to the pathophysiology of COPD and its exacerbations [12], the regulating mechanism of the expression of IL-33 in airway epithelial cells remains largely unknown.

Reactive oxygen species (ROS), including hydrogen peroxide (H_2_O_2_) derived from cigarette smoke or released by inflammatory cells, are reported to contribute to the pathogenesis of COPD and its exacerbations [13, 14]. In fact, oxidative stress markers such as H_2_O_2_ are elevated in the airways of COPD patients [15–18], and higher amounts of H_2_O_2_ are detected in the airways during the exacerbations than in those in a stable condition [19, 20]. Pretreatment with H_2_O_2_ has been reported to augment the production of pro-inflammatory cytokines and chemokines in airway epithelial cells and inflammatory cells [21, 22]. Our previous report has also demonstrated that H_2_O_2_ augments viral-derived double-stranded RNA (dsRNA)-stimulated CXC motif chemokine ligand (CXCL) 8 release in human airway epithelial cells via the activation of Toll-like receptor 3 (TLR3) signal pathway [23]. Concerning IL-33, an antioxidant, *N*-acetylcysteine (NAC) or glutathione (GSH), has been reported to inhibit the *alternaria* extract-augmented IL-33 release in an allergic mouse model, which suggests the possible involvement of oxidative stress in the regulation of IL-33 production [24]. However, it has not been determined whether oxidative stress affects the expression of IL-33 and which signal pathways are participated in the regulating mechanisms in airway epithelial cells.

The present study, therefore, was designed to determine, using H_2_O_2_ and an experimental viral infection model, the following: (1) whether oxidative stress potentiates IL-33 expression in human airway epithelial cells and which signal pathways participate in the regulating mechanisms; (2) whether oxidative stress augments IL-33 expression in the dsRNA-treated or viral infected cells; and (3) whether antioxidant treatment decreases the expression of IL-33 in airway epithelial cells from COPD patients.

## Methods

### Materials

Poly (I:C) (polyinosinic-polycytidylic acid, sodium salt, double-stranded), SB203580, SP600125, U0126, SC-514 and BAY 11-7085 were purchased from Calbiochem (La Jolla, CA). H_2_O_2_, NAC and 3-(4,5-dimethylthiazol-2-yl)-2,5-diphenylterazolium bromide (MTT) were from Sigma-Aldrich (St. Louis, MO).

### Patients

Five never smokers and six former smokers with COPD took part in our study after giving written informed consent (Table 1). COPD was diagnosed according to the GOLD guidelines [25]. All subjects had undergone surgery for lung cancer after receiving pulmonary function tests. Human bronchial tissues were obtained from the 2-4 bronchi of the lobe resected at surgery, avoiding areas involved by tumors. The tissues were used for the culture of human bronchial cells. All experiments in the study were approved by the ethics committee of Tohoku University Graduate School of Medicine.

**Table 1:** Characteristics of healthy subjects and patients with COPD

	Healthy subjects	COPD
Number	5	6
Male/Female	1/4	5/1
Age (years)	68.0 ± 3.4	68.0 ± 3.8
Ex-smoker/current smoker	0/0	6/0
Pack years	N/A	45.6 ± 8.5**
Lung function		
FEV_1_/FVC (%)	86.8 ± 3.3	62.7 ± 3.0***
FEV_1_% of predicted (%)	108 ± 6.7	75.5 ± 6.7**
DLCO/VA (%)	114 ± 4.2	85.2 ± 3.7***

Data are presented as mean ± SEM

**, *** represents, p < 0.01, p < 0.001 vs. healthy subjects

*Abbreviations*: *COPD* chronic obstructive pulmonary disease, *FEV*_*1*_ forced expiratory volume in 1 s, *FVC* forced vital capacity, *DLCO/VA* corrected carbon monoxide diffusing capacity by alveolar volume, *N/A* not applicable

### Preparation of epithelial cells

NCI-H292 cells, a human pulmonary mucoepidermoid carcinoma cell line (ATCC, Manassas, VA) and primary human bronchial epithelial cells (HBECs), acquired from lobes resected from patients at surgery, the details of which are shown in Table 1, were cultured as previously described [26]. Cells were grown to 80% confluence in culture plates and incubated in fetal bovine serum (FBS)- or growth factor-free medium for 24 h before treatment.

To investigate the effect of H_2_O_2_ on the expression of IL-33, the cells were harvested at 4 h, unless indicated otherwise, after treatment with H_2_O_2_ and stored at − 80 °C until the measurement. To examine the effect of H_2_O_2_ on the poly (I:C) or rhinovirus infection-induced IL-33 expression, H_2_O_2_ was added to the media 30 min before the treatment with poly (I:C) or rhinovirus [23]. To evaluate the effects of inhibitors, various concentrations of NAC, p38-MAPK inhibitor (SB203580), JNK inhibitor (SP600125), ERK inhibitor (U0126), IKK-2 inhibitor (SC-514) or IκBα inhibitor (BAY11-7085) were added to the media 1 h before H_2_O_2_ treatment [27, 28]. In the control group, the cells were treated with medium in the absence of H_2_O_2_, poly (I:C), or inhibitors.

### Rhinovirus infection

A stock solution of type 14 rhinovirus (RV14) [1.0 × 10^7^ tissue culture infectious dose (TCID_50_)/ ml] was obtained from a patient with a common cold and the rate of RV14 release was quantified in the same manner as previously described in methods [29]. NCI-H292 cells in culture plates were infected with RV14 at a multiplicity of infection (MOI) of 1 for 90 min in RPMI-1640 medium at 33 °C before the virus was removed and replaced with RPMI-1640 medium [26, 30, 31]. NCI-H292 cells were treated with H_2_O_2_ or vehicle 30 min prior to RV14 infection. Cells were infected with RV14 for 90 min and the virus was removed and replaced with medium containing 200 μM H_2_O_2_ or vehicle. In some experiments, cells were pretreated with NAC 1 h prior to infection. After the cells were incubated for 1 to 48 h at 33 °C, the whole cell extractions were harvested and stored at − 80 °C until required.

### Cell viability assay

Cell viability in NCI-H292 cells were evaluated by use of the MTT assay and in HBECs were evaluated by the lactate dehydrogenase (LDH) activity using Cytotoxicity Detection KitPLUS (Sigma-Aldrich) as previously described [26]. Cell viability was calculated as the percentage of viable cells among vehicle-treated cells.

### Detection of mitogen-activated protein kinase (MAPK) phosphorylation by immunoblotting

Cells were seeded in 6-well plates at a density of 1 × 10^5^/ml. At 80% confluence, and maintained in FBS-free medium for 24 h before stimulation. To evaluate the inhibitory effect of NAC on the expression of phospho-p38 (p-p38), c-jun N-terminal kinase (p-JNK) and Extracellular Signal-regulated Kinase 1/2 (p-ERK1/2), the cells were treated with NAC 60 min prior to H_2_O_2_ stimulation. After 15 min H_2_O_2_ stimulation, cells were washed with ice-cold HANK’s balanced salt solution (HBSS) and homogenized in cell lysis buffer (0.05% TritonX, 35 mM Tris-HCl, pH 7.4, 0.4 mM EGTA, 10 mM MgCl_2_, 1 μM phenylmethylsulfonyl fluoride, 100 μg/ml aprotinin and 1 μg/ml leupeptin) at 4 °C. Samples were solubilized in sodium dodecyl sulfate-polyacrylamide gels (SDS-PAGE) sample buffer. Equal amounts of protein were loaded and the separated by electrophoresis on 12.5% SDS-PAGE. After electrophoresis, the separated proteins were blotted on a PolyVinylidene DiFluoride (PVDF) membrane (Bio-Rad Laboratories, Herculer, CA). Rabbit monoclonal anti-phospho-p38 MAPK antibody, rabbit monoclonal anti-p38 MAPK antibody, rabbit monoclonal anti-phospho-JNK antibody, rabbit monoclonal anti-JNK antibody, rabbit polyclonal anti-phospho-ERK1/2 antibody, rabbit polyclonal anti-ERK1/2 antibody (1:1000 dilution, all from Cell Signaling Technology, Danvers, MA) and mouse monoclonal anti–β-actin antibody (1:10000 dilution; Sigma) were used to detect the target proteins. Appropriate peroxidase-conjugated secondary antibodies were used. Binding antibodies were detected using ECL-prime (Amersham Biosciences, Buckinghamshire, UK) and visualized with a chemiluminescence imaging system (LAS-4000 mini; Fujifilm, Tokyo, Japan). Each band intensity was quantified by densitometry (Quantity One software, Bio-Rad).

### Detection of IL-33 by nuclear extraction and immunoblotting

Cells were treated with 200 μM H_2_O_2_ for 0 to 12 h. After washing with HBSS, cells were homogenized in cell lysis buffer to obtain the nuclear fraction using Nuclear Extraction Kit (Active Motif, Carlsbad, CA) according to the manufacturer’s instructions. The following separation, blotting, detection and visualization procedures were performed in the same manner as for MAPK phosphorylation immunoblotting. Target proteins were detected with mouse monoclonal anti-IL-33 antibody (1:1000 dilution, Enzo Life Sciences, Exeter, UK) or mouse monoclonal anti-lamin A/C antibody (1:400 dilution; Santa Cruz Biotechnology, Santa Cruz, CA).

### Real-time polymerase chain reaction (PCR)

Total RNA was isolated from the cells to prepare cDNA using High capacity of RNA to cDNA kit (Applied Biosystems, Foster City, CA) according to the manufacturer’s instructions. cDNA was amplified with specific primers to *IL-33* and *glyceraldehyde-3-phosphate dehydrogenase* (*GAPDH*) using the 7500 Real-Time PCR System (Applied Biosystems). Primers used to amplify cDNA were from TaqMan Gene Expression Assays (Applied Biosystems): *IL-33* (catalogue number Hs00369211_m1), *GAPDH* (Hs99999905_ml). Relative quantification of different transcripts was determined with the comparative threshold cycle (CT) method using *GAPDH* as the endogenous control. Results were calculated as fold change over control.

### Statistical analysis

Data are expressed as the mean ± SEM. GraphPad Prism (GraphPad Software Inc., SanDiego, CA) was used to perform statistical tests. Experiments with multiple comparisons were evaluated using one way analysis of variance (ANOVA) by Bonferroni’s test to adjust for multiple comparisons. Wilcoxon matched-pairs signed rank test or Mann-Whitney U test was used for single comparison. Significance values were defined as *p* < 0.05.

## Results

### Effect of H_2_O_2_ on the expression of IL-33 in NCI-H292 cells

**Fig. 1:**
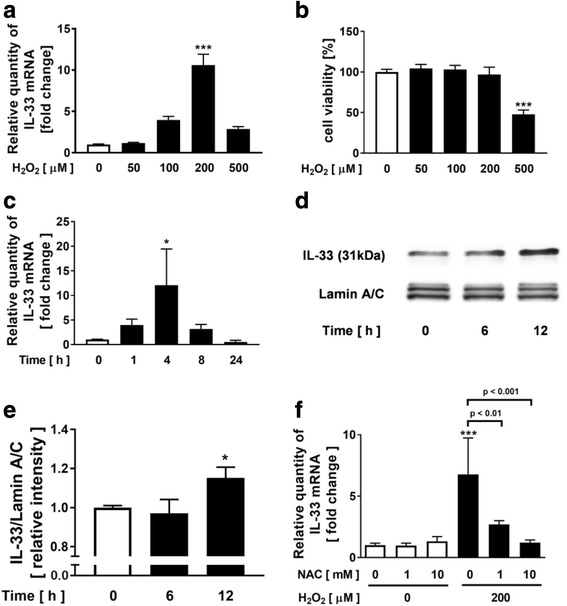
Effect of H_2_O_2_ on the expression of Interleukin-33 (IL-33) in NCI-H292 cells. **a-c** Effect of H_2_O_2_ on the gene expression of IL-33 and the cell viability in NCI-H292 cells. **a, b** Cells were treated with various concentration of H_2_O_2_. After 4 h incubation, whole cells were harvested and assayed for the gene expression of IL-33 (**a**) and cell viability (**b**). **c** Cells were treated with 200 μM H_2_O_2_. At various time points after the incubation, whole cells were harvested and assayed. **d, e** Evaluation of nuclear fraction of IL-33 protein. At various time points after the treatment with 200 μM H_2_O_2_, the nuclear fraction of cell lysates was obtained. **d** IL-33 protein in the nuclear fraction was evaluated by immunoblotting. **e** Each band intensity was assessed by densitometry. Relative intensity was calculated by dividing the IL-33 band intensity by each appropriate lamin A/C band intensity. **f** Effect of *N*-acethylcysteine (NAC) on H_2_O_2_-augmented IL-33 expression. Values are the mean ± SEM (*n* = 3 - 5). *p < 0.05, ***p < 0.001 compared with the values of vehicle-treated cells

NCI-H292 cells, a human pulmonary mucoepidermoid carcinoma cell line, have been used for evaluation of the pathophysiology in obstructive airway disease, especially as a model for mucin production, which is one of the main features of COPD [26, 27]. NCI-H292 cells share key components of various signaling pathways including MAPK and nuclear factor-kappa B (NF-κB) with normal HBECs and have been used to evaluate the involvement of oxidative stress in cytokine release and mucus secretion [27, 32, 33]. Here, we used NCI-H292 cells to clarify the involvement of oxidative stress in the expression of IL-33 in the airway epithelial cells and we investigated the effect of H_2_O_2_ on the expression of IL-33 in NCI-H292 cells. Treatment with H_2_O_2_ significantly increased the expression of IL-33 at concentrations of 0 to 200 μM in a concentration-dependent manner without affecting the cell viability (Fig. 1a-b). In addition, the H_2_O_2_-potentiated IL-33 expression reached the peak at 4 h and the potentiated expression decreased at 24 h to the same as that at 0 h (Fig. 1c). We also confirmed that H_2_O_2_ significantly increased the concentration of IL-33 protein in the compartment of nuclei in the treated cells at 12 h (Fig. 1d-e). To clarify the involvement of oxidative stress in H_2_O_2_-potentiated IL-33 expression, the effect of NAC was evaluated. Pretreatment with 1 to 10 mM NAC significantly decreased the H_2_O_2_-potentiated IL-33 expression in a concentration-dependent manner (Fig. 1f).

### Involvement of MAPK (p38, JNK, ERK1/2) and NF-κB signaling pathways in H_2_O_2_-potentiated IL-33 expression

**Fig. 2:**
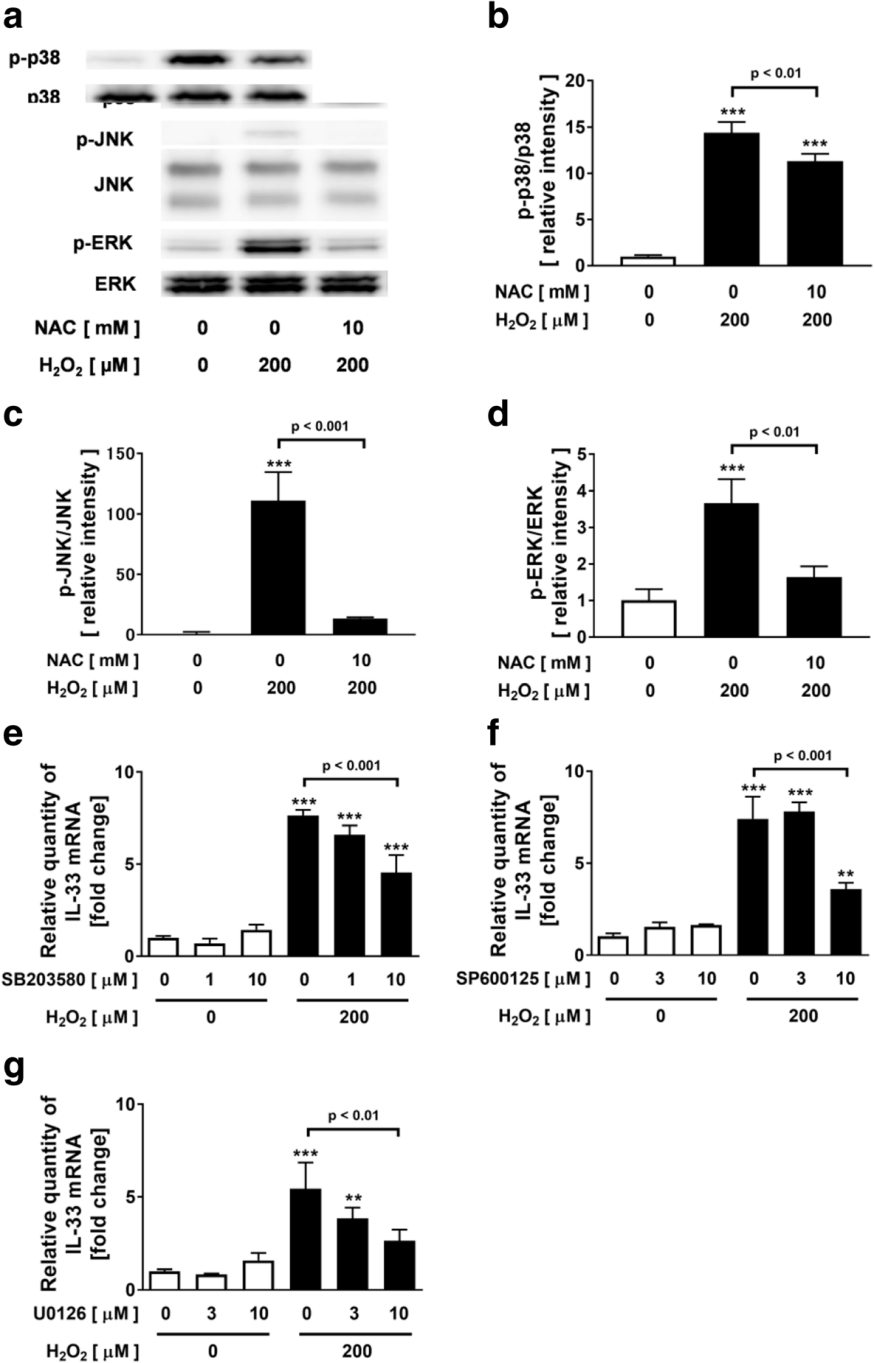
Involvement of mitogen-activated protein kinase signaling pathways in H_2_O_2_-potentiated IL-33 expression. **a-d** Effect of H_2_O_2_ on the phosphorylation of MAPK (p38, JNK, ERK1/2) in NCI-H292 cells. Cells were treated with NAC or vehicle prior to treatment with H_2_O_2_. After 15 min, whole cell lysates were obtained. **a** Phosphorylation of p38, JNK or ERK was evaluated with immunoblotting. **b-d** Band intensities were assessed by densitometry. Relative intensity was calculated by dividing the phosphorylated p38, JNK or ERK1/2 band intensity by the p38, JNK or ERK1/2 band intensity and the results were indicated as fold change over control. **e-g** Effect of p38-MAPK inhibitor (SB203580) (**e**), JNK inhibitor (SP600125) (**f**) or ERK inhibitor (U0126) (**g**) on H_2_O_2_-potentiated IL-33 expression in NCI-H292 cells. Values are the mean ± SEM (n = 3). **p < 0.01, ***p < 0.001 compared with the values of vehicle-treated cells

Previous reports indicated that oxidative stress induced the activation of MAPK [p38 MAPK, JNK, ERK1/2] and NF-κB [23, 28, 34]. Therefore, we investigated whether the potentiated effect of H_2_O_2_ on IL-33 expression is mediated by MAPK and NF-κB signaling pathways in NCI-H292 cells. Treatment with H_2_O_2_ significantly increased the phosphorylation of p38-MAPK, JNK and ERK1/2 (Fig. 2a). The H_2_O_2_-augmented phosphorylation of MAPK was inhibited by pretreatment with 10 mM NAC (Fig. 2a-d). Pretreatment with MAPK inhibitor, a p38 MAPK inhibitor, SB203580, a JNK inhibitor, SP600125, or an ERK1/2 inhibitor, U0126, inhibited the phosphorylation of p38-MAPK, JNK and ERK1/2 respectively (Additional file 1: Figure S1A-C) and dose-dependently inhibited the H_2_O_2_-augmented IL-33 mRNA expression in the cells without affecting the cell viability (Fig. 2e-g). On the other hand, pretreatment with NF-κB inhibitor (an IKK-2 inhibitor, SC-514, or an IκBα inhibitor, BAY 11-7085) did not inhibit the H_2_O_2_-augmented IL-33 expression (Additional file 1: Figure S2A-B).

### Effect of H_2_O_2_ on a synthetic viral dsRNA analogue, poly (I:C)- or viral-potentiated IL-33 expression

**Fig. 3:**
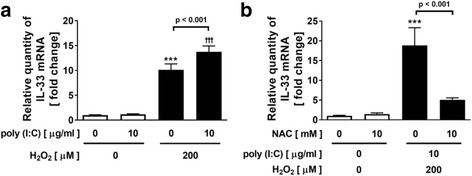
Effect of H_2_O_2_ on a synthetic viral dsRNA analogue, poly (I:C)-potentiated IL-33 expression. **a** NCI-H292 cells were treated with H_2_O_2_ or vehicle 30 min prior to the treatment with poly (I:C). After 4 h, whole cells were harvested and assayed for the IL-33 gene expression. **b** Effect of NAC on H_2_O_2_-augmented IL-33 expression in poly (I:C)-treated cells. Cells were added with NAC before H_2_O_2_ treatment, and then treated with poly (I:C). Values are the mean ± SEM (n = 3 - 4). ***p < 0.001 compared with the values of vehicle-treated control. ^†††^p < 0.001 compared with the values of poly (I:C)-treated control

**Fig. 4:**
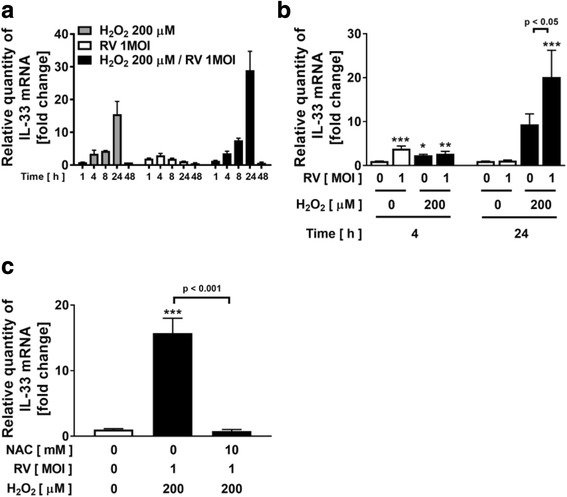
Effect of H_2_O_2_ on viral-potentiated IL-33 expression. Time course (**a**) or comparison at 4 and 24 h (**b**) of IL-33 expression after treatment with H_2_O_2_ and human rhinovirus, RV14 infection. NCI-H292 cells were infected with RV14 in the presence of H_2_O_2_ or vehicle. At various time points after incubation (**a**) or after 4 and 24 h (**b**), whole cells were harvested and assayed for the IL-33 gene expression. **c** Effect of NAC on H_2_O_2_-augmented IL-33 expression in RV14-treated cells. Cells were added with NAC before H_2_O_2_ treatment, and then infected with RV14. After 24 h RV14 infection, whole cells were harvested and assayed. Values are the mean ± SEM (*n* = 2 - 5). *p < 0.05, **p < 0.01, ***p < 0.001 compared with the values of vehicle-treated cells

Rhinovirus infection is thought to be a key factor and an important modulator of COPD exacerbations [35, 36]. In our previous study, we confirmed that TLR3 was expressed in NCI-H292 cells, and poly (I:C), which is a synthetic viral dsRNA analogue and a TLR3 ligand to mimic viral infection, activated TLR3 [26, 27]. To determine whether oxidative stress augments IL-33 expression during viral infection, we investigated the effect of H_2_O_2_ on the expression of IL-33 in poly (I:C)-treated NCI-H292 cells. H_2_O_2_ slightly but significantly augmented the expression of IL-33 in the poly (I:C)-treated cells (Fig. 3a). The augmentation was significantly inhibited by 10 mM NAC treatment (Fig. 3b). To confirm that the augmented effect observed with poly (I:C) could be replicated by a virus, we performed further experiments using human rhinovirus, RV14. RV14 infection slightly but significantly increased the expression of IL-33 in NCI-H292 cells after 4 h RV14 treatment (Fig. 4a-b). In the experimental protocol for viral infection, 200 μM H_2_O_2_ was added two times to medium 30 min prior to RV14 treatment and after 90 min incubation with RV14 to remove the RV14 from the supernatant and replace the medium. In this condition, H_2_O_2_ -potentiated IL-33 mRNA expression reached a peak at 24 h, but not at 4 h, at which point H_2_O_2_ single treatment caused the peak in Fig. 1c (Fig. 4a). Treatment with H_2_O_2_ potentiated the expression of IL-33 in the RV14-infected cells and the H_2_O_2_-potentiated IL-33 expression reached a peak at 24 h (Fig. 4a). After 24 h RV14 infection, H_2_O_2_ significantly augmented IL-33 expression in RV14-infected cells (Fig. 4b). In addition, the augmentation was significantly inhibited by 10 mM NAC treatment (Fig. 4c).

### Involvement of oxidative stress in the expression of IL-33 in HBECs from COPD patients

**Fig. 5:**
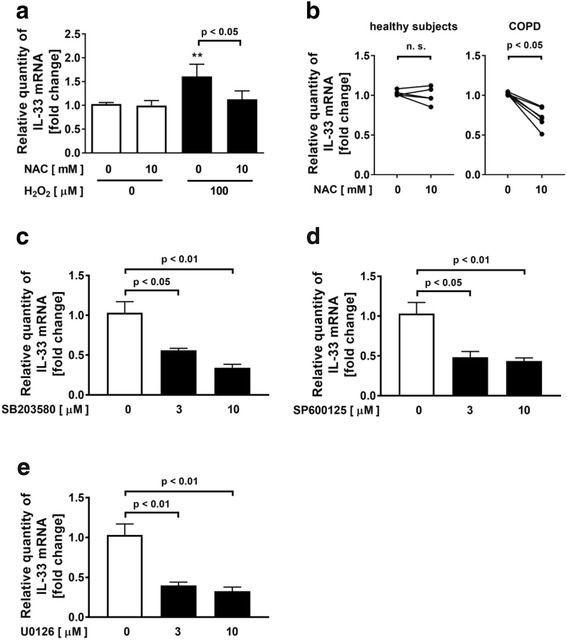
Involvement of oxidative stress in the expression of IL-33 in HBECs from COPD patients. **a** Effect of H_2_O_2_-potentiated IL-33 expression and reversal by NAC in human bronchial epithelial cells (HBECs) from healthy subjects. Cells were treated with NAC or vehicle prior to treatment with H_2_O_2_. After 4 h, whole cells were harvested and assayed for the IL-33 gene expression. **b** Effect of NAC on the expression of IL-33 in HBECs from healthy subjects and COPD patients. Cells were treated with NAC or vehicle. After 4 h, whole cells were harvested and assayed. The data are expressed as the mean ± SEM of five healthy subjects or six COPD patients. **c-e** Effect of p38-MAPK inhibitor (SB203580), JNK inhibitor (SP600125) or ERK inhibitor (U0126) on the expression of IL-33 in HBECs from COPD patients. Cells were treated with the MAPK inhibitor or vehicle. After 4 h, whole cells were harvested and assayed. The data is a representative of two independent experiments with four samples performed using HBECs from two COPD patients. **p < 0.01 compared with the values of vehicle-treated cells

In HBECs from healthy subjects, H_2_O_2_ also significantly increased the expression of IL-33 and the H_2_O_2_-augmented IL-33 expression was inhibited by pretreatment with 10 mM NAC (Fig. 5a). To clarify the involvement of oxidative stress in the expression of IL-33 in the airway epithelial cells from COPD patients, we examined the effect of an antioxidant, NAC on the expression of IL-33 in HBECs from healthy subjects and age-matched COPD patients. Under the condition without H_2_O_2_-stimulation, pretreatment with 10 mM NAC significantly decreased the expression of IL-33 in HBECs from COPD patients, but not in those from healthy subjects (Fig. 5b). On the other hand, concerning the basal expression of IL-33 in the airway epithelial cells, there was no increase in the expression of IL-33 in HBECs from COPD patients compared to those from healthy subjects (data not shown). In addition, to clarify the involvement of MAPK signaling pathways in the expression of IL-33 in HBECs from COPD patients, we examined the effect of MAPK inhibitor, a p38 MAPK inhibitor, SB203580, a JNK inhibitor, SP600125 and an ERK1/2 inhibitor, U0126, on the expression of IL-33 in HBECs from COPD patients. Under the condition without H_2_O_2_-stimulation, pretreatment with SB203580, SP600125 or U0126, significantly decreased the expression of IL-33 in HBECs from COPD patients (Fig. 5c-e).

### Cell viability

The effects of the treatments on cell viability were assessed with the MTT assay in NCI-H292 cells and with the LDH assay in HBECs. Cell viability in the NCI-H292 cells was more than 77.2% after the treatment with H_2_O_2_, excluding the concentration at 500 μM, poly (I:C), rhinovirus infection and the inhibitors. In the HBECs, cell viability was more than 79.8% after the treatment with H_2_O_2_ and NAC.

## Discussion

**Fig. 6:**
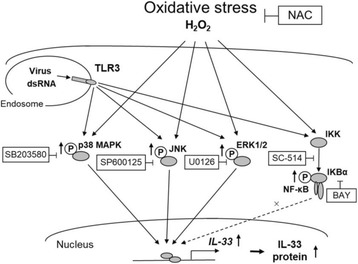
Schematic representation of the effect of oxidative stress on IL-33 expression in airway epithelial cells. H_2_O_2_ potentiates the expression of IL-33 in human airway epithelial cells. H_2_O_2_ enhances the phosphorylation of MAPK (p38, JNK, ERK1/2) and a p38 MAPK inhibitor, SB203580, a JNK inhibitor, SP600125, and an ERK1/2 inhibitor, U0126 inhibits the expression of IL-33. Neither an IKK-2 inhibitor, SC-514 nor an IκBα inhibitor, BAY11-7085 affects the H_2_O_2_-augmented IL-33 expression. H_2_O_2_ potentiates the expression of IL-33 stimulated by TLR3 ligand, poly (I:C) and rhinoviral-infection, and the potentiation is inhibited by the antioxidant NAC. These data suggest that oxidative stress enhances the expression of IL-33 via MAPK pathway and the expression of IL-33 can be further increased in human airway epithelial cells during virus infections. NAC = *N*-acetylcysteine. BAY = BAY 11-7085

In the current study, we demonstrated that H_2_O_2_ potentiated the expression of IL-33 in NCI-H292 cells and primary HBECs and clarified the involvement of MAPK signal pathway by showing that inhibition of p38 MAPK, JNK or ERK1/2 reversed the H_2_O_2_-augmented IL-33 expression. We also showed that pretreatment with H_2_O_2_ augmented the expression of IL-33 in a TLR3 ligand, poly (I:C)-treated cells and rhinovirus-infected cells. These results suggested that oxidative stress is involved in the expression of IL-33 in airway epithelial cells via MAPK signal pathway and the expression of IL-33 could be further increased in the airway epithelial cells during virus infections, which is a main cause of COPD exacerbations (Fig. 6). In addition, we demonstrated that NAC treatment decreased the expression of IL-33 in HBECs from COPD patients, which suggested that oxidative stress was involved in the expression of IL-33 in HBECs from these patients. Although a previous study suggested the possible involvement of oxidative stress in the regulation of IL-33 production in an allergic mouse model [24], here, we demonstrated that exogenous H_2_O_2_ treatment potentiates the expression of IL-33 in human airway epithelial cells via MAPK signaling. In addition, we demonstrated the possibility that oxidative stress augments the expression of IL-33 during viral infection and is involved in the expression of IL-33 in HBECs from COPD patients. Modulation of this pathway could be a therapeutic target for viral-induced exacerbations of COPD.

In the present study, we showed that exogenous H_2_O_2_ treatment potentiates the expression of IL-33 in NCI-H292 cells and primary HBECs and the potentiation is reversed by treatment with an antioxidant, NAC. In addition, we demonstrated that NAC treatment decreased the expression of IL-33 in HBECs from COPD patients, but not in those from healthy subjects. These results suggest that oxidative stress is involved in the expression of IL-33 in airway epithelial cells. Previously, IL-33 has been reported to be upregulated in human airway epithelial cells by cigarette smoke exposure [11, 37]. As cigarette smoke contains ROS including H_2_O_2_ [38, 39], cigarette smoke-potentiated IL-33 expression is consistent with the current findings. In the present study, we also demonstrated that oxidative stress potentiates the amount of IL-33 protein in the nucleus in airway epithelial cells. In a previous study, Uchida M, et al. demonstrated that an antioxidant, NAC or GSH, inhibits the *alternaria* extract-augmented IL-33 release in an allergic mice model, and they also demonstrated that NAC or GSH prevents extracellular secretion of IL-33 from IL-33-overexpressed human bronchial epithelial cells exposed to *alternaria* extract [24]. These reports support our current findings that the expression of IL-33 is regulated by oxidative stress.

H_2_O_2_ has been reported to activate not only the MAPK signal pathway, but also the NF-κB signal pathway in various cells including airway epithelial cells [23, 28, 34]. In the present study, we clarified the involvement of MAPK signal pathway in the mechanism of H_2_O_2_-augmented IL-33 expression in airway epithelial cells by showing the inhibitory effect of p38 MAPK, JNK or ERK1/2 inhibitors. In addition, we demonstrated that these inhibitors also decreased the expression of IL-33 in HBECs from COPD patients, which suggest that MAPK signaling is involved in the regulation of IL-33 expression in airway epithelial cells in COPD. Using murine alveolar epithelial cells or macrophage cells, the involvement of the MAPK signal pathway has been demonstrated in the IL-6 group of cytokines, oncostatin M- or respiratory syncytial virus infection-augmented IL-33 expression and production [40, 41]. These reports support our current results. On the other hand, in our present study, pretreatment with NF-κB inhibitors (an IKK-2 inhibitor, SC-514, or an IκBα inhibitor, BAY 11-7085) did not inhibit the H_2_O_2_-augmented IL-33 expression, suggesting that the NF-κB signal pathway is not involved in the mechanism of H_2_O_2_-augmented IL-33 expression in airway epithelial cells. In murine mast cells, IκB-α inhibitor (BAY11-7085) did not also inhibit immunoglobulin E (IgE) activation-induced IL-33 expression [42], which is consistent with our results. However, in human corneal epithelial cells, cardiac cells or murine bone marrow-derived dendritic cells, IκB-α inhibitor (BAY11-7082) and NF-κB inhibitor (quinazoline or dimethylfumarate) was reported to block TLR ligands-, tumor necrosis factor alpha (TNF-α) or ragweed pollen-potentiated IL-33 expression and production [43–45]. These reports are inconsistent with our present results. This discrepancy might result from differences in the stimulating substance, in the cell types used or in the experimental conditions including the inhibitor used.

In the present study, we showed that combination treatment of H_2_O_2_ and a synthetic viral dsRNA analogue and a TLR3 ligand, poly (I:C) or rhinovirus-infection significantly increased the expression of IL-33 compared with each treatment alone. Although the molecular mechanisms by which H_2_O_2_ potentiates poly (I:C)- or rhinovirus-induced IL-33 expression remain uncertain, previous reports have proposed possible mechanisms. TLR3 stimulation or rhinovirus infection has been demonstrated to increase the expression of IL-33 in human airway and corneal epithelial cells, and smooth muscle cells [43, 46, 47]. In addition, the MAPK signal pathway has been reported to be activated by TLR3 stimulation or rhinovirus infection in human airway epithelial cells [28, 48, 49]. In our previous studies, we demonstrated that oxidative stress enhances the expression of TLR3, and that cigarette smoke potentiates TLR3-ERK signal pathway in human airway epithelial cells [23, 27]. These results suggest that the MAPK signal pathway is involved in the H_2_O_2_-potentiated IL-33 expression induced by poly (I:C) or rhinovirus infection. Recently, Hristova M, et al. have reported that nicotinamide adenine dinucleotide phosphate (NADPH) oxidase homolog, dual oxidase 1 (DUOX1)-derived H_2_O_2_, is involved in the *alternaria*-induced IL-33 release in airway epithelial cells. Activated DUOX1-induced H_2_O_2_ has been shown to stimulate A disintegrin and metalloproteinase 17 (ADAM17), which is responsible for the shedding of EGFR pro-ligand and the activation, and leads to the activation of the EGFR-ERK signal pathway [50]. These reports may suggest that oxidative stress augments the rhinovirus-stimulated IL-33 expression via TLR3-EGFR signal pathway. Further studies are necessary to prove this hypothesis.

In the comparison of the expression of IL-33 in the disease status of COPD, there was no increase in the expression of IL-33 in HBECs from COPD patients compared to HBECs from healthy subjects, which is inconsistent with a result previously reported that higher expression of IL-33 appeared in the airway epithelial cells from patients with COPD [10]. However, this higher expression of IL-33 is shown in severe COPD in GOLD stage IV. Another group has also reported that an increase of the IL-33 protein level in the whole lung appears only from severe COPD in GOLD stage III / IV [11]. In our current study, all of the patients evaluated were mild COPD in GOLD stage I-II, which might explain the discrepancy. In the current study, we also showed that NAC treatment decreased the expression of IL-33 in HBECs from COPD patients, but not in those from healthy subjects, suggesting that oxidative stress is involved in the expression of IL-33 in airway epithelial cells in patients with COPD. We have previously reported an increased level of oxidative stress in HBECs of COPD patients compared to those in healthy subjects [51] and, therefore, it could be thought that NAC treatment decreased the increased level of oxidative stress in the HBECs of COPD patients, which affects the expression of IL-33. However, there remains the discrepancy that an increased level of oxidative stress is suggested in the HBECs from COPD patients and NAC treatment decreased the IL-33 expression from the basal levels only in COPD patients, but there is no increase in the expression of IL-33 in the HBECs from COPD patients compared to healthy subjects. Several negative regulations of IL-33 expression have been demonstrated, such as by lipopolysaccharide treatment via TLR signaling in NCI-H292 cells, or by one of short noncoding RNAs, microRNA-487b in human monocytes [52, 53]. These unknown negative regulatory mechanisms might affect the expression of IL-33 in the airway epithelial cells of COPD patients. Further studies are needed to clarify this point.

As a limitation of the present study concerning HBECs, there is a possibility that sex differences and smoking status might affect the results because HBECs were derived from 5 female healthy subjects and 1 female COPD patient. In addition, all of the healthy subjects were non-smokers, whereas all of the COPD patients were ex-smokers. Also, in the experiments in HBECs from COPD patients and healthy subjects, the evaluation of IL-33 expression was performed by measuring the levels of mRNA expression and was not confirmed by examining the protein expression.

## Conclusions

We demonstrated that oxidative stress is involved in the expression of IL-33 in airway epithelial cells via the MAPK signal pathway and that it augments IL-33 expression during viral infection. This mechanism may participate in the regulation of IL-33 expression in airway epithelial cells in COPD and in viral-induced exacerbations. Modulation of this pathway could be a therapeutic target for viral-induced exacerbations of COPD.

## Additional file

Additional file 1: **Figure S1.** Effect of mitogen-activated protein kinase (MAPK) inhibitors in H_2_O_2_-potentiated phosphorylation of MAPK (p38, JNK, ERK1/2). **Figure S2.** Effect of NF-κB inhibitors in H_2_O_2_-potentiated IL-33 expression. (PDF 449 kb)

## Abbreviations

ADAM17: A disintegrin and metalloproteinase 17
ANOVA: Analysis of variance
COPD: Chronic obstructive pulmonary disease
CXCL8: CXC motif chemokine ligand 8
DLCO: Diffusing capacity of the lung for carbon monoxide
dsRNA: Double-stranded RNA
DUOX1: Dual oxidase 1
EGFR: Epidermal growth factor receptor
ERK: Extracellular signal-regulated kinase
FBS: Fetal bovine serum
FEV_1_: Forced expiratory volume in one second
FVC: Forced vital capacity
GAPDH: Glyceraldehyde-3-phosphate dehydrogenase
GOLD: The global initiative for chronic obstructive lung disease
GSH: Glutathione
H_2_O_2_: Hydrogen peroxide
HBECs: Human bronchial epithelial cells
HBSS: HANK’s balanced salt solution
IgE: Immunoglobulin E
IL-33: Interleukin-33
JNK: c-jun N-terminal kinase
LDH: Lactate dehydrogenase
MAPK: Mitogen-activated protein kinase
MOI: Multiplicity of infection
MTT: 3-(4,5-dimethylthiazol-2-yl)-2,5-diphenylterazolium bromide
NAC: *N*-acetylcysteine
NADPH: Nicotinamide adenine dinucleotide phosphate
NF-κB: Nuclear factor-kappa B
PCR: Polymerase chain reaction
poly (I:C): Polyinosinic-polycytidylic acid
PVDF: PolyVinylidene DiFluoride
ROS: Reactive oxygen species
RV14: Type 14 rhinovirus
SDS-PAGE: Sodium dodecyl sulfate- polyacrylamide gels
SEM: Standard error of the mean
Th2: T helper 2 cell
TLRs: Toll-like receptors
TNF-α: Tumor necrosis factor alpha
VA: Alveolar volume
