# Correction to: Oxidative stress enhances the expression of IL-33 in human airway epithelial cells

**DOI:** 10.1186/s12931-018-0817-9

**Published:** 2018-06-12

**Authors:** Hiroyuki Aizawa, Akira Koarai, Yutaka Shishikura, Satoru Yanagisawa, Mutsuo Yamaya, Hisatoshi Sugiura, Tadahisa Numakura, Mitsuhiro Yamada, Tomohiro Ichikawa, Naoya Fujino, Masafumi Noda, Yoshinori Okada, Masakazu Ichinose

**Affiliations:** 1 Department of Respiratory Medicine, Tohoku University Graduate School of Medicine, 1-1 Seiryo-machi, Aoba-ku, Sendai, 980-8574 Japan; 2 Department of Advanced Preventive Medicine for Infectious Disease, Tohoku University Graduate School of Medicine, 1-1 Seiryo-machi, Aoba-ku, Sendai, 980-8575 Japan; 3 Department of Thoracic Surgery, Institute of Development, Aging and Cancer, Tohoku University, 4-1 Seiryo-machi, Aoba-ku, Sendai, 980-8575 Japan

## Correction

Figure 2 of this original publication was incorrectly formatted. The updated Fig. 2 is published in this correction article [1].

**ᅟ:**
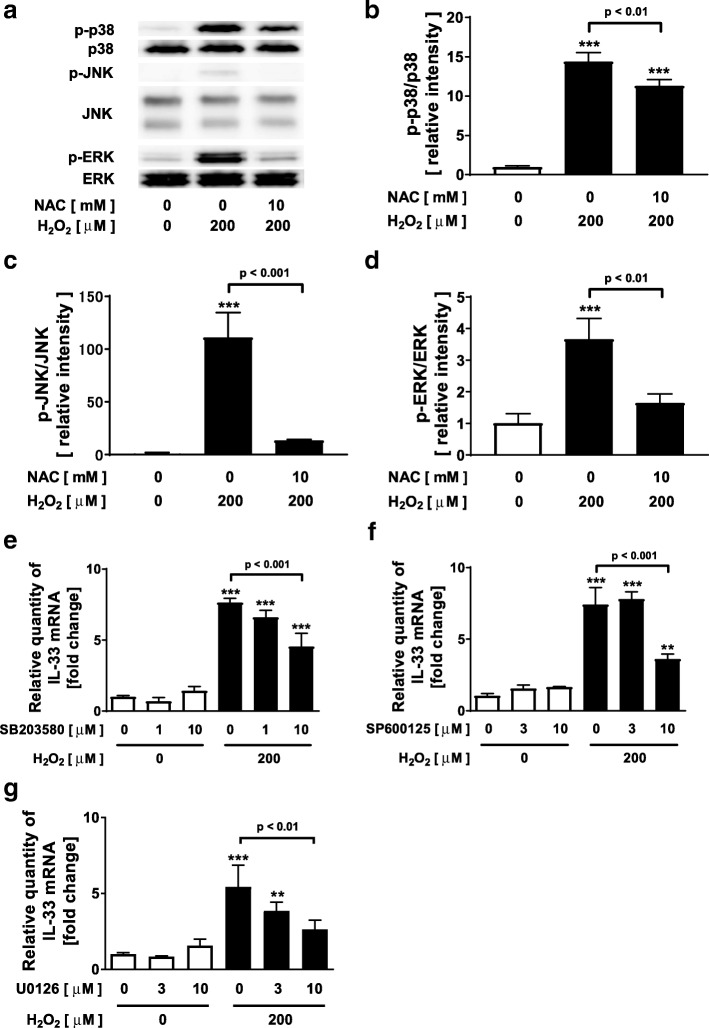
ᅟ
