# Supplementary material for: Oxidative stress enhances the expression of IL-33 in human airway epithelial cells
Source: Respir Res. 2018 Mar 27;19:52. doi: 10.1186/s12931-018-0752-9 (PMC5872512; doi:10.1186/s12931-018-0752-9)
Supplement: Supplementary file 1 — Figure S1. Effect of mitogen-activated protein kinase (MAPK) inhibitors in H2O2-potentiated phosphorylation of MAPK (p38, JNK, ERK1/2). Figure S2. Effect of NF-κB inhibitors in H2O2-potentiated IL-33 expression. (PDF 449 kb) [file 12931_2018_752_MOESM1_ESM.pdf]

Oxidative stress enhances the expression of IL-33 in human airway epithelial cells

Hiroyuki Aizawa, M.D.[1], Akira Koarai, M.D., Ph.D.[1], Yutaka Shishikura, M.D., Ph.D.[1], Satoru Yanagisawa, M.D., Ph.D. [1], Mutsuo Yamaya, M.D., Ph.D.[2], Hisatoshi Sugiura, M.D., Ph.D.[1], Tadahisa Numakura, M.D., Ph.D.[1], Mitsuhiro Yamada, M.D., Ph.D.[1], Tomohiro Ichikawa M.D., Ph.D.[1], Naoya Fujino M.D., Ph.D.[1], Masafumi Noda M.D., Ph.D.[3], Yoshinori Okada, M.D., Ph.D.[3] and Masakazu Ichinose, M.D., Ph.D.[1]

[1] Department of Respiratory Medicine, Tohoku University Graduate School of Medicine, 1-1 Seiryo-machi, Aoba-ku, Sendai 980-8574, Japan.

[2] Department of Advanced Preventive Medicine for Infectious Disease, Tohoku University Graduate School of Medicine, 1-1 Seiryo-machi, Aoba-ku, Sendai 980-8575, Japan.

[3] Department of Thoracic Surgery, Institute of Development, Aging and Cancer, Tohoku University, 4-1 Seiryo-machi, Aoba-ku, Sendai 980-8575, Japan.

Additional file

Figure legends

Figure S1. Effect of mitogen-activated protein kinase (MAPK) inhibitors in H_2O_2-potentiated phosphorylation of MAPK (p38, JNK, ERK1/2). The phosphorylation of p38, JNK and ERK was evaluated with immunoblotting in NCI-H292 cells. Pretreatment with MAPK inhibitor, p38 MAPK inhibitor (SB203580), JNK inhibitor (SP600125) or ERK1/2 inhibitor (U0126) inhibited the phosphorylation of p38-MAPK (A), JNK (B) and ERK1/2 (C) respectively. The data are representative of three independent experiments.

Figure S2. Effect of NF-κB inhibitors in H_2O_2-potentiated IL-33 expression. (A, B) IKK-2 inhibitor (SC-514) or IκBα inhibitor (BAY11-7085) was added 1 h before H_2O_2 treatment of NCI-H292 cells. After 4 h, whole cells were harvested and assayed for the IL-33 gene expression by real-time Polymerase Chain Reaction (PCR). Values are the mean ± SEM (n = 3). ***$p < 0.001$ compared with the values of vehicle-treated cells.

Figure S1

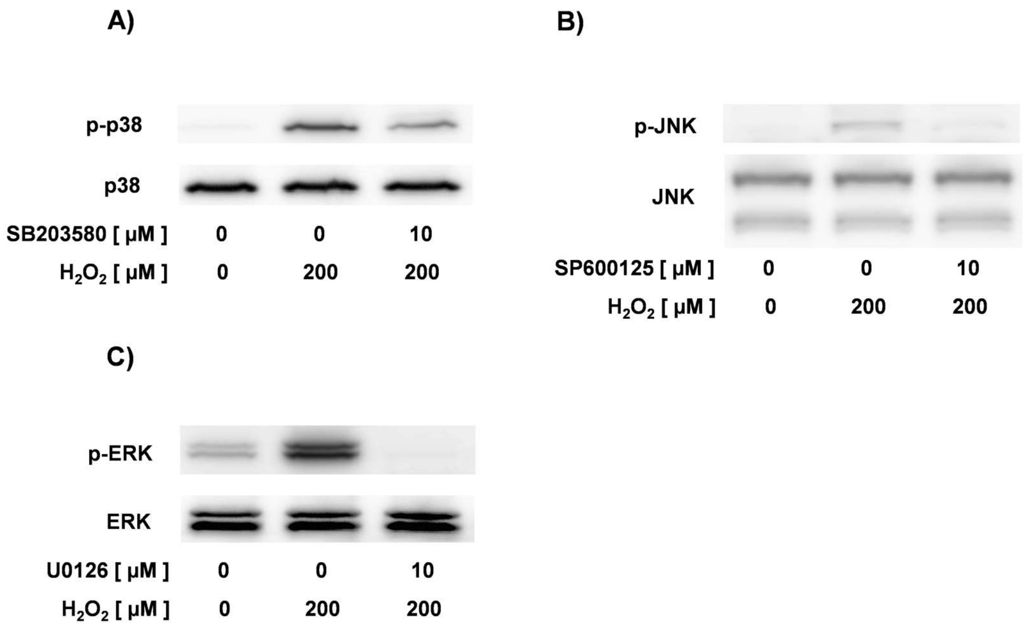
A)
p-p38
p38
SB203580 [μM] 0 0 10
H2O2 [μM] 0 200 200
B)
p-JNK
JNK
SP600125 [μM] 0 0 10
H2O2 [μM] 0 200 200
C)
p-ERK
ERK
U0126 [μM] 0 0 10
H2O2 [μM] 0 200 200

Figure S2

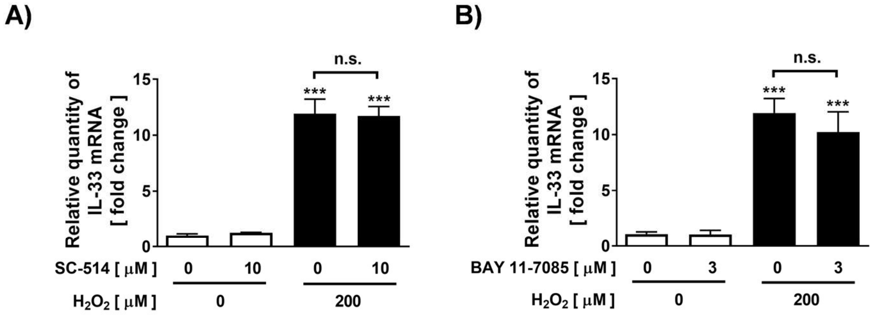
A)
B)
Relative quantity of IL-33 mRNA [fold change]
n.s.
***
15
10
5
0
SC-514 [μM]
0
10
H2O2 [μM]
200
BAY 11-7085 [μM]
3
